# Genetic Etiologies in Developmental and/or Epileptic Encephalopathy With Electrical Status Epilepticus During Sleep: Cohort Study

**DOI:** 10.3389/fgene.2021.607965

**Published:** 2021-04-08

**Authors:** Pan Gong, Jiao Xue, Xianru Jiao, Yuehua Zhang, Zhixian Yang

**Affiliations:** Department of Pediatrics, Peking University First Hospital, Beijing, China

**Keywords:** electrical status epilepticus during sleep, encephalopathy, genetic, etiology, epilepsy

## Abstract

**Background:**

Recently, the electroencephalogram pattern of electrical status epilepticus during sleep (ESES) had been reported in some genetic disorders, and most of them were noted with developmental and epileptic encephalopathy (DEE) or epileptic encephalopathy (EE). This study aimed to determine the genetic etiologies and clinical characteristics of ESES in DEE/EE.

**Methods:**

We performed a cohort study in cases of DEE or EE with ESES. Tio-based genetic testing was performed in 74 cases and was analyzed to identify underlying variants.

**Results:**

Pathogenic or likely pathogenic variants were identified in 17/74 cases, including *KCNQ2* (*n* = 6), *KCNA2* (*n* = 5), *GRIN2A* (*n* = 3), *SLC9A6* (*n* = 1), *HIVEP2* (*n* = 1), and *RARS2* (*n* = 1). Eleven were boys. The median age at seizure onset was 6 months. ESES occurred at the mean age of 2.0 ± 1.2 years, predominant in the Rolandic region in 14 years. Twelve of 17 cases had the first stage of different epilepsy preceding ESES: 2/12 were diagnosed as Ohtahara syndrome, 2/12 were diagnosed as infantile spasms, 3/12 were diagnosed as DEE, and 5/12 were diagnosed as EE without the epileptic syndrome.

**Conclusion:**

Monogenic variants explained over 20% of DEE/EE with ESES. ESES could be an age-related feature in genetic disorders and occurred after the first stage of different epilepsy. Both age-related factors and genetic etiology were suggested to play a role in the occurrence of ESES in genetic DEE/EE.

## Introduction

Electrical status epilepticus during sleep (ESES) initially described an electroencephalogram (EEG) pattern where interictal focal or multifocal spike-and-wave occupied at least 85% of the EEG tracing during non-rapid eye movement (NREM) sleep (Patry et al., 1971). It was now also used as a cutoff value ranging from 50 to 85% in the literatures (Loddenkemper et al., 2011; Sánchez Fernández et al., 2012). The widely recognized ESES-related syndromes included benign childhood epilepsy with centrotemporal spikes, atypical benign partial epilepsy (ABPE), Landau–Kleffner syndrome, and epileptic encephalopathy with continuous spike-and-wave during sleep (CSWS). The latter three were known as epileptic encephalopathy (EE) (Loddenkemper et al., 2011). A few cases and small series suggested some degree of genetic predisposition in the EEG pattern of ESES (Sánchez Fernández et al., 2012). Recently, ESES had been reported in some genetic disorders, such as *KCNQ2*, *ZEB2*, and *SLC9A6* (Bonanni et al., 2017; Lee et al., 2017; Mathieu et al., 2018). Masnada et al. (2017) described an ESES-like pattern in *KCNA2*-related developmental and epileptic encephalopathy (DEE). In 2018, a systematic review on all reported genetic etiologies of ESES-related syndrome summarized 11 monogenic variants identified in 60 cases (Kessi et al., 2018). Most of the reported patients were noted with EE or DEE. Here, we aimed to describe the clinical and genetic characteristics of patients with ESES and EE or DEE caused by monogenic variants.

## Materials and Methods

### Ethical Approval of the Study Protocol

This study was approved by the Ethical Committee of Peking University First Hospital, and written informed consents were obtained from the legal guardians (parents) of the subjects for publication.

### Participants

Firstly, the EEG database over the past 3 years at the Pediatric Department of Peking University First Hospital was queried for ESES. For the purpose of this study, the spike-wave index (SWI) during NREM sleep of ESES was demarcated as above 50%. Secondly, patients with ESES recognized from the first step were screened by the following clinical criteria: (a) seizures, (b) having a developmental delay from birth or suffering from remarkable developmental impairments after seizure onset, and (c) having taken a clinical or a research-based genetic assessment, including trio-based targeted gene panels testing and whole-exome sequencing, (d) before the ESES occurrence, EEG pattern at the early stage of epilepsy was especially included in our study, including burst suppression, multifocal discharges, hypsarrhythmia, and normality. Patients with acquired structural lesions and those diagnosed with Rett syndrome and autism were excluded. We reviewed history obtained from medical records and from families *via* phone and/or written survey, including sex, age at seizure onset, seizure types, perinatal and personal history, family history, neurological development, magnetic resonance imaging findings, treatment, and other relevant clinical data. Pediatric epileptologists reviewed series of primary EEG recordings of each patient. Reports were reviewed when original tracings were not available.

### Pathogenicity Analysis

**FIGURE 1:**
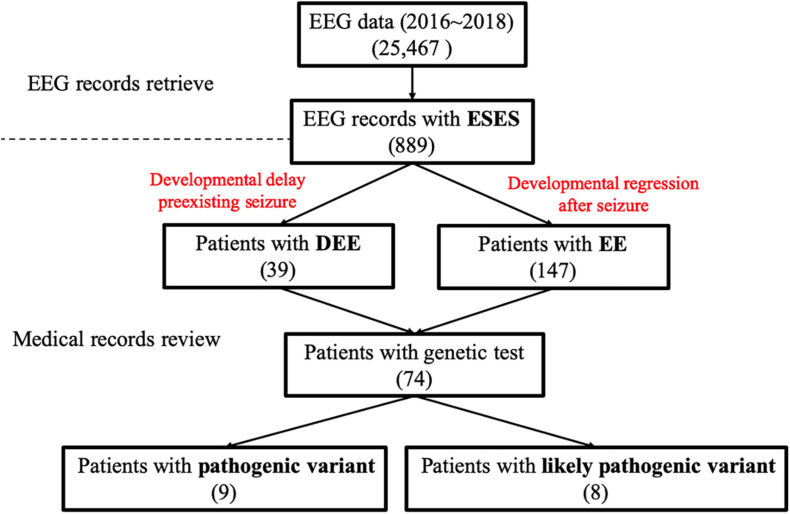
Data screening flow chart. EEG, electroencephalography; DEE, developmental and epileptic encephalopathy; EE, epileptic encephalopathy.

We described variants using human genome 19 (hg19) coordinates. We used inheritance pattern, *in silico* predictions, control database (including the Exome Aggregation Consortium, Exon Variant Server, 1000 Genomes database, and Single Nucleotide Polymorphism Database), clinical laboratory reports, and findings in the literature to assess pathogenicity. For the prediction of the pathogenicity of nonsynonymous variants, we used Polyphen2^1^ and SIFT^2^. For available original data, after assessing the known epilepsy genes (Supplementary Table 1), we considered other candidate genes, among which we prioritized *de novo* heterozygous, compound heterozygous, or homozygous variants in genes with a plausible role in epilepsy. Variants considered pathogenic or likely pathogenic were verified by Sanger sequencing. For available genetic testing reports, the clinical significance of the identified variants was interpreted according to the guidelines set out by the American College of Medical Genetics (Richards et al., 2015). A complete description of the methods can be found in Figure 1.

## Results

Seventy-four cases of DEE/EE with ESES had undergone genetic testing. Forty-five were boys. The median age at seizure onset was 29.4 months (range 1 day to 108 months), whereas ESES occurrence was 5.3 years (range 1.1–13 years). The median age at the last available medical records was 6.8 years (range 1.9–14 years). Eventually, a positive result was identified in 17/74 (23.0%): six with a likely pathogenic *KCNQ2* variant, five with a pathogenic *KCNA2* variant, three with a pathogenic or likely pathogenic *GRIN2A* variant, one with a pathogenic *SLC9A6* variant, one with a pathogenic *HIVEP2* variant, and one with likely pathogenic *RARS2* variants (Table 1). Eleven of 17 were boys. The median age at seizure onset was 6 months (range 1 day to 52 months). The mean age on the occurrence of ESES was 2.0 ± 1.2 years. The SWI of ESES achieved above 85% in 13 and 50–85% in 4. Cases were followed till a mean age of 5.1 ± 2.4 years. Main clinical data are defined in Table 2.

**TABLE 1:** Summary of monogenic variants for a series of 17 cases of DEE/EE with ESES.

ID	Epilepsy gene	Method(s)of identification	Suspected or known pathogenic mutation	Zygosity	Inheritance pattern	Phenotype	*In silico* predictions	ExAC database	Pathogenicity
1	*KCNQ2*	Clinical gene panel	c.736G>C (p.Ala246Pro)	Heterozygous	*De novo*	IS → ESES without syndrome	PP-2: 0.919 SIFT: 0	Not found	Likely pathogenic
2	*KCNQ2*	Clinical gene panel	c.998G>A (p.Arg333Gln)	Heterozygous	paternal	DEE without syndrome → ABPE	PP-2: 0.999 SIFT: 0.003	Not found	Likely pathogenic
3	*KCNQ2*	Clinical gene panel	c.920T>C (p.Leu307Pro)	Heterozygous	*De novo*	OS → ESES without syndrome	PP-2: 1.000 SIFT: 0	Not found	Likely pathogenic
4	*KCNQ2*	Clinical gene panel	c.832A>T (p.Ile278Phe)	Heterozygous	*De novo*	OS → ESES without syndrome	PP-2: 0.959 SIFT: 0	Not found	Likely pathogenic
5	*KCNQ2*	Clinical gene panel	c.740C>T (p.Ser247Leu)	Heterozygous	*De novo*	DEE without syndrome → ESES without epilepsy	PP-2: 0.678 SIFT: 0	Not found	Likely pathogenic
6	*KCNQ2*	Clinical gene panel	c.952C>G (p.Leu318Val)	Heterozygous	*De novo*	IS → ESES without epilepsy	PP-2: 0.998 SIFT: 0.001	Not found	Likely pathogenic
7	*KCNA2*	Clinical WES	c.1214C>T (p.Pro405Leu)	Heterozygous	paternal	EE without syndrome → ABPE	PP-2: 1.000 SIFT: 0	Not found	Pathogenic
8	*KCNA2*	Clinical WES	c.1214C>T (p.Pro405Leu)	Heterozygous	*De novo*	EE without syndrome → ABPE	PP-2: 1.000 SIFT: 0	Not found	Pathogenic
9	*KCNA2*	Clinical gene panel	c.1214C>T (p.Pro405Leu)	Heterozygous	*De novo*	EE without syndrome → ABPE	PP-2: 1.000 SIFT: 0	Not found	Pathogenic
10	*KCNA2*	Research exome	c.1214C>T (p.Pro405Leu)	Heterozygous	*De novo*	EE without syndrome → ABPE	PP-2: 1.000 SIFT: 0	Not found	Pathogenic
11	*KCNA2*	Clinical gene panel	c.1214C>T (p.Pro405Leu)	Heterozygous	*De novo*	EE without syndrome → ABPE	PP-2: 1.000 SIFT: 0	Not found	Pathogenic
12	*GRIN2A*	Clinical gene panel	c.1034delG (p.Gly345Alafs*19)	Heterozygous	*De novo*	ABPE	N/A	Not found	Pathogenic
13	*GRIN2A*	Clinical WES	c.2107C>T (p.Gln703*)	Heterozygous	*De novo*	ABPE	N/A	Not found	Pathogenic
14	*GRIN2A*	Clinical WES	c.1592C>T (p.Thr531Met)	Heterozygous	*De novo*	ABPE	PP-2: 1.000 SIFT: 0	Not found	Likely pathogenic
15	*SLC9A6*	Clinical gene panel	c.1178_1180del (p.394del)	Hemizygous	X-linked	CSWS	N/A	Not found	Pathogenic
16	*HIVEP2*	Clinical WES	c.5935C>T (p.Arg1979*)	Heterozygous	*De novo*	ABPE	N/A	Not found	Pathogenic
17	*RARS2*	Clinical WES	c.[991A>G; 1718C>T] p.[Ile331Val; Thr573Ile]	Compound heterozygous	AR	DEE without syndrome → ABPE	PP-2: 0.670 SIFT: 0.013	Not found	Likely pathogenic

DEE, development and epileptic encephalopathy; EE, epileptic encephalopathy; ESES, epileptic encephalopathy with electrical status epilepticus during sleep; IS, infantile spasms; PP-2, PolyPhen2; ABPE, epileptic encephalopathy of atypical benign partial epilepsy; OS, Ohtahara syndrome; WES, whole exome sequencing; CSWS, epileptic encephalopathy with continuous spike-and-wave during sleep; AR, autosomal recessive. The symbol * is used to indicate a translation termination (stop) codon.

**TABLE 2:** Clinical characteristics of 17 cases of genetic DEE/EE with ESES.

Cases	Case 1	Case 2	Case 3	Case 4	Case 5	Case 6
Gender, age	M, 2 y 4 m	M, 8 y 9 m	F, 1 y 11 m	F, 2 y 1 m	M, 4 y 10 m	M, 4 y 4 m
Gene, mutations	KCNQ2 c.736G>C (p.Ala246Pro) *de novo*	KCNQ2 c.998G>A (p.Arg333Gln) paternal	KCNQ2 c.920T>C (p.Leu307Pro) *de novo*	KCNQ2 c.832A>T (p.Ile278Phe) *de novo*	KCNQ2 c.740C>T (p.Ser247Leu) *de novo*	KCNQ2 c.952C>G (p.Leu318Val) *de novo*
Age at epilepsy onset/seizure type	1 d/focal seizure	2 d/GTCS	2 d/tonic seizure	3 d/tonic seizure	2 d/focal seizure	5 m/spasm
Other seizure types	Spasm, AA ± SE	Clonic seizure, focal seizure during sleep	Spasm, focal seizure, FS+SE	Focal seizure	FS, spasm	MC
Seizure outcome	Seizure controlled 2 m–1 y 7 m. breakthrough AA ± SE at age 1 y 7 m	Seizure controlled since 8 y 1 m	Uncontrolled	Uncontrolled	Seizure controlled 6 m–4 y 10 m. breakthrough spasms at age 4 y 10 m	Seizure free since 10 m (controlled by adrenocorticotrophic hormone)
EEG at onset	Hypsarrhythmia	Multifocal SW and Sh-W	Burst suppression	Burst suppression	Multifocal SW and Sh-W	Hypsarrhythmia
EEG in the evolution (age at ESES onset)	GSW with Ar or Pr predominance SWI 85–100% (1 y 7 m)	Sp and SW in Rolandic region SWI 85–90% (3 y 5 m–7 y 1 m)	SW and polySp in Pr regions SWI 75–85% (1 y 11 m)	Multifocal SW and Sh-W with Rolandic predominance SWI 95% (1 y 1 m)	Sp and Sh-W in Rolandic region SWI > 85% (1 y 10 m–4 y 10 m)	EEG normalized 1 y–1 y 10 m Sp and Sh-W in Rolandic region SWI>50% (2 y 3 m)
Current AEDs	TPM, VGB, LTG	VPA, CZP, LTG	LEV, VPA	VPA, TPM	LEV, TPM, OXC	–
Development before seizure onset	Delayed from birth	Delayed from birth	Delayed from birth	Delayed from birth	Delayed from birth	Delayed from birth
Neurological features after seizure onset	Psychom.dev. delay Psychom.dev. improved after focal seizures and spasms controlled	Psychom.dev. delay Severe language delay Psychom.dev. improved after seizure free	Psychom.dev. delay Poor visual contact, head deviation to one side	Psychom.dev. delay Poor visual contact, head deviation to one side	Psychom.dev. delay Psychom.dev. improved after seizure free 6 m–4 y 10 m	Psychom.dev. regression Language delay, impairment of fine motor skills
Imaging	Cerebral atrophy	Delayed myelination	Diffuse cortical dysplasia	Diffuse cortical dysplasia	Agenesis of the corpus callosum	Agenesis of the corpus callosum
Additional features	–	Family history of epilepsy	–	Hemolysis neonatorum	–	Test tube baby (twins) Pathologic jaundice

*EEG, electroencephalogram; AEDs, anti-epileptic drugs; M, male; y, years; m, months; d, days; AA, atypical absence; SE, status epilepticus; GSW, generalized spike and waves; Ar, anterior; Pr, posterior; ESES, electrical status epilepticus during sleep; SWI, spike-wave index; TPM, topiramate; VGB, vigabatrin; LTG, lamotrigine; Psychom.dev., psychomotor developmental; GTCS, generalized tonic–clonic seizure; SW, spike-waves; Sh-W, sharp-waves; Sp, spikes; VPA, valproic acid; CZP, clonazepam; F, female; FS, febrile seizure; polySp, polyspikes; LEV, levetiracetam; MC, myoclonus.*						

Cases	Case 7	Case 8	Case 9	Case 10	Case 11	
Gender, age	F, 8 y 11 m	M, 7 y 7 m	M, 2 y 11 m	M, 4 y 4 m	F, 2 y 8 m	
Gene, mutations	KCNA2 c.1214C>T (p.Pro405Leu) paternal	KCNA2 c.1214C>T (p.Pro405Leu) *de novo*	KCNA2 c.1214C>T (p.Pro405Leu) *de novo*	KCNA2 c.1214C>T (p.Pro405Leu) *de novo*	KCNA2 c.1214C>T (p.Pro405Leu) *de novo*	
Age at epilepsy onset/seizure type	11 m/Febrile tonic–clonic seizure	6 m/focal seizure	8 m/focal seizure	10 m/MC	5 m/afebrile tonic–clonic seizure	
Other seizure types	Focal seizures during sleep, SE, ENM	ENM, FS	AA ± atonic, febrile SE	focal seizure during sleep	MC, SE, FS, focal seizures during sleep	
Seizure outcome	Seizure controlled since 6 y 8 m	Focal seizures controlled since 2 y 8 m, ENM controlled since 5 y 8 m	Uncontrolled	Uncontrolled	Uncontrolled	
EEG at onset	N	N	GSW with Pr predominance	GSW with Pr predominance	GSW with Pr predominance	
EEG in the evolution (age at ESES onset)	Multifocal SW and Sh-W with Rolandic predominance SWI 60–85% (3 y 9 m)	Sp and Sh-W in Rolandic region SWI 60–85% (5 y 4 m)	Sp and Sh-W in Rolandic region, GSW SWI > 85% (2 y 5 m)	Sp and Sh-W in Rolandic region ± GSW SWI 60–85% (2 y 11 m)	Sp and Sh-W in Rolandic region SWI 70% (2 y 7 m)	
Current AEDs	VPA, CZP	VPA, LEV, CLB	VPA, CZP, LEV	VPA, LEV	VPA, LEV	
Development before seizure onset	N	N	N	N	N	
Neurological features after seizure onset	Psychom.dev. regression Severe language delay, impairment of fine motor skills, Psychom.dev. improved after seizure free	Psychom.dev. regression Severe language delay, impairment of fine motor skills, tremor, Psychom.dev. improved after seizure free	Psychom.dev. regression Language delay	Psychom.dev. regression Language delay	Psychom.dev. regression Language delay	
Imaging	N	N	N	N	N	
Additional features	Family history of epilepsy	Neonatal asphyxia	Family history of epilepsy	–	–	

*EEG, electroencephalogram; AEDs, anti-epileptic drugs; F, female; y, years; m, months; SE, status epilepticus; ENM, epileptic negative myoclonus; N, normal; SW, spike-waves; Sh-W, sharp-waves; ESES, electrical status epilepticus during sleep; SWI, spike-wave index; VPA, valproic acid; CZP, clonazepam; Psychom.dev., psychomotor developmental; M, male; FS, febrile seizures; Sp, spikes; LEV, levetiracetam; CLB, clobazam; AA, atypical absence; GSW, generalized spike and waves; Pr, posterior; MC, myoclonic seizures.*						

Cases	Case 12	Case 13	Case 14	Case 15	Case 16	Case 17
Gender, age	F, 5 y 6 m	M, 4 y 6 m	F, 6 y 5 m	M, 8 y	M, 7 y 10 m	M, 3 y 6 m
Gene, mutations	GRIN2A c.1034delG (p.Gly345Alafs*19) *de novo*	GRIN2A c.2107C>T (p.Gln703*) *de novo*	GRIN2A c.1592C>T (p.Thr531Met) *de novo*	SLC9A6 c.1178_1180del (p.394del) *de novo*	HIVEP2 c.5935C>T (p.Arg1979*) *de novo*	RARS2 c.[991A>G; 1718C>T] (p.[Thr573Ile; Ile331Val]) *de novo*
Age at epilepsy onset/seizure type	2 y 4 m/focal seizure during sleep	3 y 6 m/focal seizure during sleep	3 y 10 m/focal seizure during sleep ± sGTCS	1 y 11 m/focal seizure	4 y 4 m/febrile GTCS	3 m/MC
Other seizure types	AA, MC	–	SE, FS	Febrile GTCS, MC, AA	Focal seizures, AA	Focal seizures, AA
Seizure outcome	Seizure controlled since 5 y	Uncontrolled	Seizure controlled since 5 y 2 m	Uncontrolled	Uncontrolled	Uncontrolled
EEG at onset	BG slowing, Multifocal Sp and Sh-W	Multifocal Sp with Rolandic predominance	Multifocal Sp and Sh-W with Pr predominance	Sp in Fr region with right predominance	Sh-W in Fz	GSW
EEG in the evolution (age at ESES onset)	BG slowing, Sp, and Sh-W in Rolandic region SWI 85–100% (4 y 6 m–5 y 6 m)	Sp and Sh-W in Rolandic region SWI 50–60% (4 y 6 m)	Sp and Sh-W in Rolandic region SWI 70% (5 y 8 m)	Sp and Sh-W in Ar region SWI 60–95% (3 y 3 m)	Multifocal Sp and Sh-W with Rolandic predominance SWI 80–100% (5 y 6 m)	Sp and Sh-W in Rolandic region SWI 85% (3 y 6 m)
Current AEDs	VPA, LEV, CZP	VPA, LEV	VPA, LEV, CZP	VPA, LEV	VPA, CZP, LEV	TPM, PB
Development before seizure onset	N	Language delay, impairment of motor skills	Language and reaction delay	Delayed from birth	Considerable global delay at 2 years old	Delayed from birth
Neurological features after seizure onset	Psychom.dev. regression Severe language and reaction delay, self-care disability	Psychom.dev. regression Severe language delay	Psychom.dev. regression Severe language delay	Psychom.dev. regression Severe language delay, independent but ataxic walking aggressiveness, hyperactivity	Psychom.dev. regression Severe language delay, impairment of gross and fine motor coordination	Psychom.dev. regression Impairment of gross and fine motor skills, language delay
Imaging	N	N	N	N	N	Cerebral atrophy; progressive white matter depletion
Additional features	–	Premature birth, neonatal asphyxia	–	Prevention of miscarriage	Broad forehead, hypotonia, malnutrition	Hypotonia, progressive microcephaly

EEG, electroencephalogram; AEDs, anti-epileptic drugs; F, female; y, years; m, months; AA, atypical absence; MC, myoclonic seizures; BG, background; Sp, spikes; Sh-W, sharp-waves; ESES, electrical status epilepticus during sleep; SWI, spike-wave index; VPA, valproic acid; LEV, levetiracetam; CZP, clonazepam; N, normal; Psychom.dev., psychomotor developmental; M, male; sGTCS, secondary generalized tonic–clonic seizure; SE, status epilepticus; FS, febrile seizures; Pr, posterior; GTCS, generalized tonic–clonic seizure; Ar, anterior; GSW, generalized spike and waves; TPM, topiramate; PB, phenobarbitone. The symbol * is used to indicate a translation termination (stop) codon.

### Genotypes and Phenotypes of the Cases With *KCNQ2* Variants

Six missense variants were identified in six cases. The variants arose *de novo* except one. Case 2 carried a paternal variant R333Q that had been reported to be associated with benign familial neonatal seizures (BFNS) (Singh et al., 2003).

**FIGURE 2:**
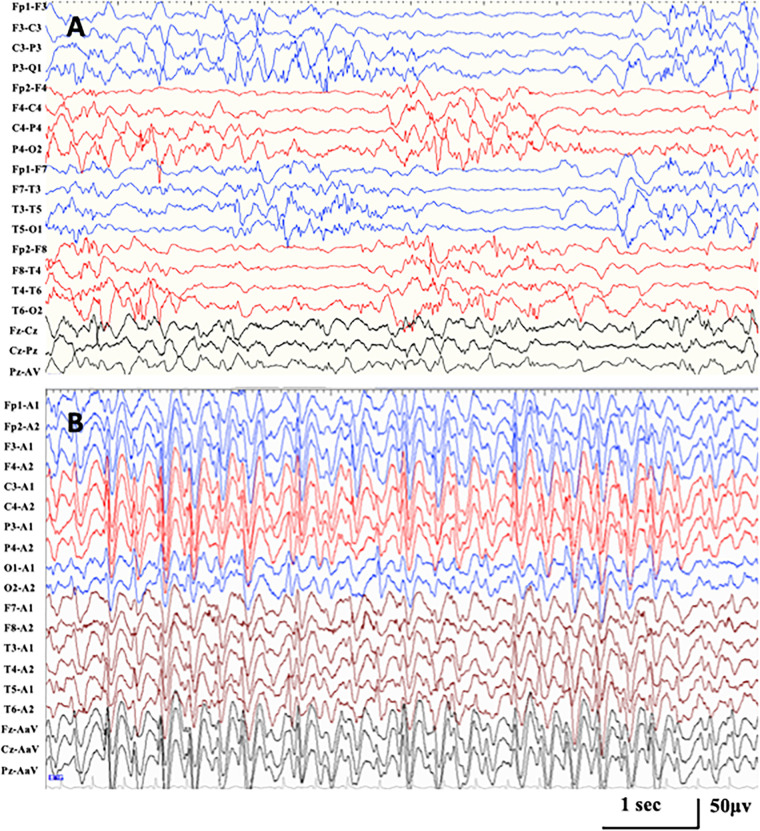
Representative EEG in case 1 with *KCNQ2-*related disorders. **(A)** Interictal EEG at seizure onset of 1 month after birth demonstrating hypsarrhythmia with high amplitude multifocal spikes and asynchronization between hemispheres and chaotic background. **(B)** Interictal EEG at the age of 1.6 years, demonstrating generalized spike-and-wave with anterior predominance during NREM sleep (SWI 85%).

All had seizure onset within the first week except one with seizure onset at 5 months (case 6). All had a global developmental delay from birth. The first stage of epilepsy in all was inconsistent with DEE. Two had onset of tonic seizure accompanied by burst suppression, diagnosed with Ohtahara syndrome (cases 3 and 4). One had onset of a focal seizure and then epileptic spasm (case 1), and one had onset of epileptic spasm (case 6), both accompanied by hypsarrhythmia, and they were diagnosed with infantile spasms. One had onset of generalized tonic–clonic seizure (GTCS) (case 2), and one had onset of the focal seizure (case 5), both accompanied by multifocal discharges, which could not be classified into an epilepsy syndrome. The distribution of epileptiform discharges shifted, and the SWI further achieved ESES at a mean age of 2.0 ± 0.7 years, which brought the disease into the second stage of epilepsy. ESES presented with Rolandic predominance in four, posterior predominance in one, anterior or posterior predominance in one (Figure 2). In cases 1, 3, and 4, combining with seizure types, they did not fit with traditional ESES-related syndrome. Case 2 with a paternal variant had nocturnal focal seizures during the second stage, diagnosed as ABPE. He achieved ESES remission at 7.1 years old and seizure-free at 8.1 years old with a remarkably developmental improvement, especially on language. His father had seizure onset at 1 month after birth and became seizure-free at 1 year. There were additional six families affected. The age at seizure onset ranged from 2 days to 6 months after birth, and all of them became seizure-free before 1 year of age. Except for case 2, all of the affected families met the diagnosis of BFNS. Cases 5 and 6 had no seizure during the occurrence of ESES. In case 5, seizures were controlled at the age of 6 months, and ESES occurred at the age of 1.8 years. However, he had breakthrough spasms at the age of 4.8 years, accompanied by multifocal and generalized epileptiform discharges. In case 6, seizures were controlled by adrenocorticotropic hormone at 10 months, and the EEG normalized. Epileptiform discharges in the Rolandic region occurred at the age of 1.8 years and evolved into ESES at 2.3 years without a seizure. Neuroimaging was abnormal in all: cerebral atrophy in one, delayed myelination in one, diffuse cortical dysplasia in two, and agenesis of the corpus callosum in two.

### Genotypes and Phenotypes of the Cases With *KCNA2* Variants

All five cases carried the same missense variant of P405L that was reported to be a loss-of-function variant (Masnada et al., 2017). The variants of four arose *de novo*, with only the remaining one having a paternal variant (case 7).

The mean age at seizure onset was 8.0 ± 2.5 months, and prior cognitive and motor development was normal in all. Seizure semiology at onset was a focal seizure in two, myoclonic seizure in one, febrile tonic–clonic seizure in one, and afebrile tonic–clonic seizure in one. Epilepsy onset was accompanied or followed by a developmental regression in all. The EEG at onset was normal in two and showed generalized spikes and waves with posterior predominance in three. All five cases were diagnosed as EE without relative epilepsy syndrome at the first stage of epilepsy. They all had nocturnal focal seizures at a mean age of 2.5 ± 0.9 years. The distribution of epileptiform discharges transformed to Rolandic region, and ESES occurred at a mean age of 3.3 ± 1.2 years. All of them were diagnosed with ABPE at the second stage of epilepsy. A paternal variant was identified in case 7, whose father also had an epilepsy history of ABPE. The age at the last follow-up ranged from 2.7 to 8.8 years. The seizure was controlled with a remarkably developmental improvement at the age of 6.8 and 5.8 years in cases 7 and 8, respectively.

### Genotypes and Phenotypes of the Cases With *GRIN2A* Variants

A frameshift variant, a nonsense variant, and a missense variant in *GRIN2A* were identified in three cases. All variants occurred *de novo*.

Two cases had a developmental delay from birth, with one of them delivered prematurely. The median age of focal seizure onset during sleep was 38.7 months (range 28–46 months). All had a developmental regression after seizure onset. ESES in the Rolandic region occurred at a median age of 4.9 years (range 4.5–5.7 years). All of them were diagnosed with ABPE. The age at the last follow-up ranged from 4.5 to 6.4 years. Two had seizures controlled at the age of 5 and 5.2 years. They both had developmental improvement but still presented with considerable developmental delay.

### Other genes, Known or Novel Association With Electrical Status Epilepticus During Sleep

A novel hemizygous *SLC9A6* variant was found in case 14. He had a delayed milestone from birth. The age of focal seizure onset during sleep was 1.9 years old with developmental regression. He developed multiple seizure types during the course, including febrile GTCS, myoclonic seizures, and atypical seizures. ESES in the anterior region was identified at 3.3 years old. He was diagnosed with CSWS. At the last follow-up of age 8 years, he presented with independent but ataxic walking, no oral speech, hyperkinetic behavior, and active seizures.

**FIGURE 3:**
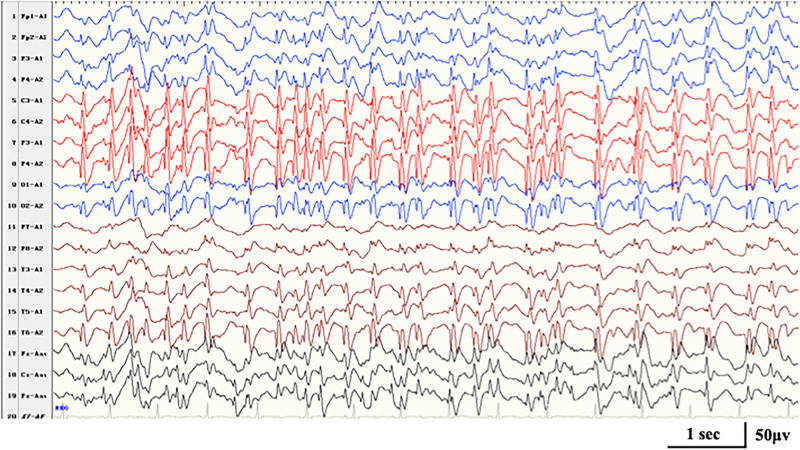
Interictal EEG in case 16 with *HIVEP2-*related disorders at the age of 7 years, demonstrating generalized spike-and-wave with Rolandic predominance during NREM sleep (SWI 90%).

A heterozygous *HIVEP2* variant was identified in case 15. The same nonsense variant had been reported (Goldsmith et al., 2019). The considerable global developmental delay became apparent at the age of 2 years. He had an onset of febrile GTCS at 4.3 years old. Then, nocturnal focal seizures and atypical absence occurred during the course. ESES with Rolandic predominance occurred at 5.6 years old (Figure 3). With a gradual developmental regression, he was diagnosed with ABPE. At the last follow-up of age 7.8 years, he had notable difficulties in gross and fine motor coordination with poor expression language skills and active seizures. Additional features include a broad forehead, hypotonia, and malnutrition.

**FIGURE 4:**
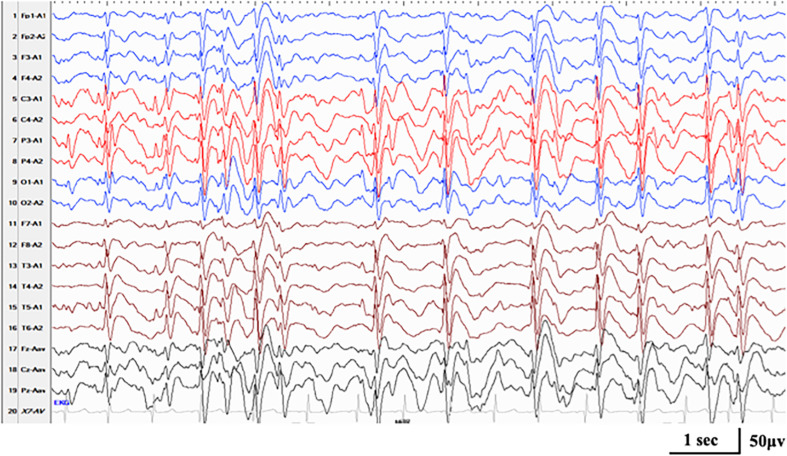
Interictal EEG in case 17 with *RARS2*-related disorders at the age of 3.5 years, demonstrating generalized spike-and-wave with Rolandic predominance during NREM sleep (SWI 85%).

Compound heterozygous variants in *RARS2* were identified in case 16. The patient had a global developmental delay since birth. He gradually presented with hypotonia, with a feeding difficulty apparent from 5 months and lethargy and progressive microcephaly from 7 months. He had frequent multifocal myoclonic seizures at 3 months with developmental regression. Neuroimaging was notable for cerebral atrophy and progressive white matter depletion. He was diagnosed as DEE without relative epilepsy syndrome at the first stage of epilepsy. During the course, he had nocturnal focal seizures and atypical seizures. ESES in the Rolandic region occurred at 3.5 years old (Figure 4). He was diagnosed with ABPE at the second stage of epilepsy. At the last follow-up of age 3.5 years, he still had active seizures and severe developmental delay.

### Electrical Status Epilepticus During Sleep in Developmental and Epileptic Encephalopathy/Epileptic Encephalopathy Caused by A Monogenic Variant

The mean age at the occurrence of ESES in 17 cases was 2.0 ± 1.2 years. ESES presented with Rolandic predominance in 14, posterior predominance in one, anterior region predominance in one, and anterior or posterior predominance in one. Twelve of 17 cases had the first stage of different epilepsy preceding ESES, and 2/12 were diagnosed as Ohtahara syndrome, 2/12 as infantile spasms, 3 as DEE, and 5/12 as EE without the special epileptic syndrome. At the second stage of epilepsy with ESES, 10/17 cases were diagnosed as ABPE, 1/17 as CSWS, 4/17 as ESES without the epileptic syndrome, and 2/17 as ESES without epilepsy.

## Discussion

Recently, an increasing number of pieces of literature had reported the occurrence of ESES in disorders caused by monogenic variants (Coppola et al., 2003; Kobayashi et al., 2006; Bonanni et al., 2017; Lee et al., 2017; Mathieu et al., 2018). A systematic review on all reported genetic etiologies of ESES-related syndrome summarized 11 monogenic variants identified in 60 cases, including *GIRN2A*, *SCN2A*, *KCNA2*, *KCNQ2*, *KCNB1*, *SLC6A1*, *SLC9A6*, *ATN1*, *SPRX2*, *CNKSR*, and *OPA3* (Kessi et al., 2018). Masnada et al. (2017) observed dramatic activation of EEG abnormalities during sleep in *KCNA2*-related DEE. They described it as an “ESES-like” pattern due to a different EEG pattern at the early stage of epilepsy and the lack of a longitudinal follow-up. For the purpose of this study, we still used the term “ESES,” referring to the EEG pattern. Here was the largest single-center study, which provided an insight into the genetic etiologies in DEE/EE with ESES and described the electroclinical characteristics in them. Pathogenic or likely pathogenic variants explained 23.0% of cases in our cohort.

Caused by the impaired function of potassium channels due to *KCNQ2* variants, *KCNQ2* encephalopathy was a clinical syndrome with a wide range from BFNS to early onset epileptic encephalopathy (Fang et al., 2019). Several affected cases with BFNS harboring *KCNQ2* variants later developing benign childhood epilepsy with centrotemporal spikes had been reported (Maihara et al., 1999; Coppola et al., 2003; Ishii et al., 2012). In our cohort, all six cases had a first stage of epilepsy with DEE and developed to the second stage with the occurrence of ESES. One with a paternal variant of R333Q was diagnosed as ABPE at the second stage. He achieved ESES remission at 7.1 years old and seizure-free at 8.1 years old with a remarkably developmental improvement, further supporting a diagnosis of ABPE. The R333Q variant had been previously reported in a family with BFNS (Singh et al., 2003). In our cohort, except for this case, the other seven affected families, including his father, were diagnosed with BFNS, whose seizure became free before the first year of life. It indicated that even the same variant could lead to a different phenotype and outcome, which could be explained by other unknown modifier genes and environmental factors. The remaining five cases did not fit with traditional ESES-related syndrome. Among them, ESES appeared almost before the age of 2 years, which was earlier than the typical onset age in traditional ESES-related syndrome. Currently, only two cases of *KCNQ2* encephalopathy with ESES had been reported (Lee et al., 2017). With the six cases added, we proposed that ESES could appear as an age-related EEG phenomenon in *KCNQ2* encephalopathy, but it might appear earlier due to genetic factors. Also, there could be a different EEG pattern at the first stage of epilepsy preceding ESES.

As a recently discovered gene associated with epilepsy disorders, more than 50 cases bearing the *KCNA2* variant had been reported (Masnada et al., 2017; Sachdev et al., 2017). Based on the severity of the encephalopathy and the seizure disorder, the phenotype associated with the *KCNA2* variant might be differentiated into two main groups, with the milder phenotype correlating with loss-of-function variants and more severe phenotype with gain-of-function variants (Sachdev et al., 2017). Totally, six cases bearing the same loss-of-function P405L variant had been reported, and five of them presented with ESES (Allou et al., 2017; Sachdev et al., 2017). Here, five cases all carried the P405L variant, and the second stage of epilepsy with ESES was in line with EE of ABPE. However, there was a different EE at the first stage. With the five cases added, it suggested that the specific variant itself in *KCNA2* was largely responsible for specific clinical symptoms. Epilepsy disorders related to the P405L variant tended to evolve from a different EE at the first stage into EE of ABPE. It indicated that both genetic and age-related factors played a role in the occurrence of ESES. The underlying mechanisms leading to ESES were not completely understood. The genetic basis might disrupt the normal maturation of neuronal networks, leading to an age-related pattern of electroclinical expression of ESES (Sánchez Fernández et al., 2012). Actually, our group has already worked on the study of the P405L mutation causing ESES, and we generated an induced pluripotent stem cell line from an EE patient with ESES carrying the *KCNA2* (p.P405L) mutation (Gong et al., 2020). The induced pluripotent stem cell line will be useful to better understand the pathogenesis of ESES and discover new targets for pharmacological intervention.

The loss-of-function variant of *GRIN2A* was previously recognized as the principal genetic cause of ESES, and it was reported that up to 20% of ESES-related syndrome had a pathogenic variant in *GRIN2A* (Pavlidis et al., 2019). Our previous study identified the *GRIN2A* variant in 4/77 cases with ESES syndrome in a Chinese cohort (one with Landau–Kleffner syndrome and three with ABPE) (Yang et al., 2018). Here, three new cases with ABPE were identified. Two of them had a developmental delay preexisting seizure onset with further developmental regression accompanied by ABPE. It suggested that *GRIN2A* variants had a close relationship with the EEG pattern of ESES, but to what extent was still uncertain. The exact cellular and molecular mechanisms linking the *GRIN2A* variant and continuous spiking in NREM sleep were not well understood, but the disturbed glutamatergic pathways signaling during the neurodevelopmental age window might play a role (Bishop et al., 2015). GluN2A subunit encoded by *GRIN2A* mainly expressed after birth, whereas other subunits encoded by other genes were at a high level at embryonic stages, modestly diminishing after birth (Monyer et al., 1994). The theory discussed earlier could explain why genes had an age-dependent effect on the development of ESES.

In 1999, the variant involved in *SLC9A6* was first described to underlie Christianson syndrome, characterized by moderate-to-severe intellectual disability with absent or very limited language development, epilepsy, ataxia, hyperkinetic behavior, and acquired microcephaly (Christianson et al., 1999). So far, ESES was reported in five cases with Christianson syndrome within the critical age window of 4–8 years (Ieda et al., 2019). In one of them, seizure-free and resolution of ESES were achieved at the age of 8 years, and transient clinical recovering of up to 15 words was noticed (Coorg and Weisenberg, 2015). In our cohort, the case was predominantly manifested with developmental impairment related to genetic variants. He was diagnosed as CSWS with ESES occurrence during the course. *HIVEP2* was first considered a rare cause of neurodevelopmental abnormalities in 2016 (Steinfeld et al., 2016). To date, *HIVEP2* variants had been published in 14 cases with the last follow-up of age 5 to 24 years, and the clinical feature was nonspecific, including hypotonia, development delay, intellectual disability, and dysmorphic features (Park et al., 2019). Only 2 of 14 cases were reported to have definite seizures (Park et al., 2019). Here, our case also had remarkable developmental delay preceding ABPE. It constituted the first known instance of ESES occurrence in *HIVEP2*-related disorder. *RARS2* was an identified cause of pontocerebellar hypoplasia type 6 with autosomal recessive inheritance, typically characterized by pontine atrophy, vermian hypoplasia, infantile encephalopathy, generalized hypotonia, and intractable seizures (Park et al., 2019). Up to now, a total of 26 cases with *RARS2* variants had been reported with the last follow-up of age 2 months to 24 years (Zhang et al., 2018). Here, we firstly described ESES associated with *RARS2*. The case was diagnosed as DEE without epilepsy syndrome at the first stage and developed to ABPE at the second stage. Our findings suggested that ESES could be an age-related feature in genetic disorders related to *SLC9A6*, *HIVEP2*, and *RARS2*. Severe developmental impairment and encephalopathy caused by genetic variant was the main manifestation and relatively mild epilepsy might occur during the course, just like Rett syndrome. Further deterioration of the cognitive and behavioral status during the course warranted a detection of the possible occurrence of ESES.

Studies reported the evolution of the SWI over time with the onset of ESES at 4–8 years of age (Loddenkemper et al., 2011). In our cohort, the mean age at the occurrence of ESES was 2.0 ± 1.2 years, earlier than the typical onset age. It might be due to the genetic factor. In some genetic disorders, especially *KCNQ2* and *KCNA2*, there was always the first stage of different EEG patterns preceding ESES. In most cases, ESES was predominant in the Rolandic region, and most of them were diagnosed as ABPE. We speculated that the epileptiform discharge in these genetic disorders usually localized or transformed to the Rolandic region with further evolution of ESES.

## Conclusion

More than 20% of DEE/EE with ESES were identified with monogenic variants, including *KCNQ2*, *KCNA2*, *GRIN2A*, *SLC9A6*, *HIVEP2*, and *RARS2*. With an age-related feature in genetic disorders, ESES could follow the first stage of different EEG patterns in precedence. Further deterioration of the cognitive and behavioral status during the course warranted a detection of the possible occurrence of ESES. Both age-related and genetic factors played a role in the occurrence of ESES in genetic DEE/EE.

## Data Availability Statement

The raw data supporting the conclusions of this article will be made available by the authors, without undue reservation.

## Ethics Statement

The studies involving human participants were reviewed and approved by the Ethical Committee of Peking University First Hospital. Written informed consent to participate in this study was provided by the participants’ legal guardian/next of kin.

## Author Contributions

ZY conceptualized and designed the study, coordinated the study overall, and revised the manuscript. PG co-designed the study, drafted the initial manuscript, and revised the manuscript. JX and XJ helped collect and summarize data and reviewed the manuscript. YZ interpreted the data and critically reviewed the manuscript. All authors contributed to the article and approved the submitted version.

## Conflict of Interest

The authors declare that the research was conducted in the absence of any commercial or financial relationships that could be construed as a potential conflict of interest.

## Footnotes

1 http://genetics.bwh.harvard.edu./pph2/

2 https://sift.bii.a-star.edu.sg/
