# Supplementary material for: Genetic Etiologies in Developmental and/or Epileptic Encephalopathy With Electrical Status Epilepticus During Sleep: Cohort Study
Source: Front Genet. 2021 Apr 8;12:607965. doi: 10.3389/fgene.2021.607965 (PMC8060571; doi:10.3389/fgene.2021.607965)
Supplement: Supplementary file 1 [file Table_1.DOC]

Gene	Gene	Gene	Gene	Gene	Gene	
AARS	ARHGEF15	CASR	COX15	DPAGT1	FOLR1	
ABAT	ARHGEF9	CC2D2A	COX6B1	DPM1	FOXG1	
ABCC2	ARL13B	CDH13	CPA6	DPM3	FOXP2	
ABCC8	ARSA	CDH9	CPLX1	DPYD	FOXRED1	
ACADSB	ARSB	CDKL5	CPS1	DRD2	FRRS1L	
ACOX1	ARSE	CEP152	CPT1A	DRD3	FUCA1	
ACTB	ARV1	CEP290	CPT2	DTNBP1	G6PD	
ACY1	ARX	CHD2	CSTB	EBP	GABBR2	
ADAM22	ASAH1	CHD7	CTNNA3	ECM1	GABRA1	
ADCY5	ASPA	CHI3L1	CTSA	EEF1A2	GABRA6	
ADGRG1	ATIC	CHRNA2	CTSD	EFHC1	GABRB1	
ADGRV1	ATN1	CHRNA3	CTSF	EHMT1	GABRB2	
ADK	ATP13A2	CHRNA4	CUL4B	EIF2B1	GABRB3	
ADSL	ATP13A4	CHRNA5	CYB5R3	EIF2B2	GABRD	
AFG3L2	ATP1A2	CHRNA7	CYBB	EIF2B3	GABRG2	
AGA	ATP1A3	CHRNB2	CYFIP2	EIF2B4	GALC	
AHI1	ATP2A2	CLCN2	CYP2R1	EIF2B5	GALNS	
AKT1	ATP5F1A	CLCN4	CYP2U1	ELP4	GAMT	
ALDH4A1	ATP6AP2	CLCNKA	D2HGDH	EMX2	GATAD2B	
ALDH5A1	ATP7A	CLCNKB	DAO	EPB41L1	GATM	
ALDH7A1	ATPAF2	CLN3	DAOA	EPHB2	GBA	
ALG1	ATRX	CLN5	DBH	EPM2A	GCDH	
ALG11	ATXN10	CLN6	DBT	ERBB4	GCK	
ALG12	B4GALT1	CLN8	DCX	ERLIN2	GCSH	
ALG13	BANK1	CNPY3	DDC	ETFA	GFAP	
ALG2	BCKDHA	CNTN5	DDOST	ETFB	GLB1	
ALG3	BCKDHB	CNTNAP2	DENND5A	ETFDH	GLDC	
ALG6	BCKDK	COA5	DEPDC5	EVC	GLRA1	
ALG8	BCS1L	COG1	DGKD	FADD	GLRB	
ALG9	BOLA3	COG4	DHCR7	FASN	GLUD1	
ALPL	BRAF	COG5	DHFR	FASTKD2	GLUL	
AMACR	BSN	COG6	DIAPH3	FBP1	GNAO1	
AMER1	BTD	COG7	DISC1	FCGR2B	GNE	
AMT	C12orf65	COG8	DLD	FGD1	GNPTAB	
AP3B2	C4A	COL18A1	DMPK	FGF12	GNPTG	
AP4S1	CACNA1A	COL1A1	DNAJC5	FGF8	GNS	
APOL2	CACNA1H	COL4A1	DNAJC6	FGFR3	GOSR2	
APOL4	CACNB4	COMT	DNASE1	FH	GPC3	
APP	CACNG2	COQ2	DNM1	FKRP	GPHN	
APTX	CAD	COQ8A	DOCK6	FKTN	GRIA3	
ARG1	CARS2	COQ9	DOCK7	FLNA	GRIN1	
ARHGAP31	CASK	COX14	DOLK	FLVCR2	GRIN2A	
 Supplemental materials
Table S1: The list of genes reported to cause different genetic forms of epilepsy. 


Gene	Gene	Gene	Gene	Gene	Gene	
GRIN2B	KCNC2	MDH2	NDUFV2	PEX14	PRRT2	
GRIN2D	KCNH1	ME2	NECAP1	PEX16	PSAP	
GRN	KCNH5	MECP2	NEDD4L	PEX19	PSAT1	
GSS	KCNJ1	MED12	NEU1	PEX2	PTCH1	
GUF1	KCNJ10	MEF2C	NF1	PEX26	PTPN11	
GUSB	KCNJ11	MFSD8	NF2	PEX3	PTPN22	
GYS1	KCNMA1	MGAT2	NGLY1	PEX5	PUS1	
HAX1	KCNQ1	MLC1	NHLRC1	PEX6	QDPR	
HCN1	KCNQ2	MMACHC	NHS	PEX7	RAB39B	
HDAC4	KCNQ3	MMUT	NID2	PGAP2	RAB3GAP1	
HEXA	KCNT1	MOCS1	NOTCH3	PGAP3	RAF1	
HEXB	KCNT2	MOCS2	NPC1	PGK1	RANBP2	
HFE	KCTD7	MOCS3	NPC2	PGM1	RARS2	
HGSNAT	KDM5C	MOGS	NPHP1	PHF6	RBFOX1	
HLA-DQA1	KIF11	MPC1	NPHS1	PHGDH	RBFOX2	
HLA-DQB1	KIF1A	MPDU1	NR3C1	PIGA	RBFOX3	
HNF1B	KMT2D	MPI	NRAS	PIGL	RELN	
HNRNPH1	KRAS	MR1	NRXN1	PIGN	RFT1	
HNRNPU	KRIT1	MTHFR	NTNG1	PIGO	RHOBTB2	
HP	L2HGDH	MTOR	NTRK2	PIGP	RNASEH2A	
HPD	LAMA2	MTR	NUBPL	PIGV	RNASEH2B	
HRAS	LARGE1	MTRR	OFD1	PIGW	RNASEH2C	
HSD17B10	LBR	NAGLU	OPA1	PIGY	ROGDI	
HSD17B4	LGI1	NDE1	OPHN1	PLA2G6	RPGRIP1L	
HTR2A	LGR4	NDN	OTC	PLCB1	RPIA	
HTT	LIAS	NDUFA1	PAFAH1B1	PLP1	RTN4R	
HYAL1	LIG4	NDUFA11	PAH	PLPBP	RYR1	
IDH2	LMX1B	NDUFA2	PAK3	PMM2	RYR3	
IDS	LRPPRC	NDUFAF1	PANK2	PNKD	SAMHD1	
IDUA	MAGI1	NDUFAF2	PAX6	PNKP	SCARB2	
IER3IP1	MAGI2	NDUFAF3	PC	PNPO	SCN1A	
IFNG	MAGT1	NDUFAF4	PCCA	POLG	SCN1B	
IL6	MAN1B1	NDUFAF5	PCDH19	POMGNT1	SCN2A	
INPP5E	MANBA	NDUFB3	PCNT	POMT1	SCN3A	
INS	MAP2K1	NDUFS1	PDHA1	POMT2	SCN4A	
INSR	MAP2K2	NDUFS2	PDHX	PPOX	SCN8A	
IQSEC2	MAPK10	NDUFS3	PDSS1	PPT1	SCN9A	
ITPA	MBD5	NDUFS4	PDSS2	PQBP1	SCO2	
KCNA1	MCCC2	NDUFS6	PEX1	PRICKLE1	SDHA	
KCNA2	MCOLN1	NDUFS7	PEX10	PRICKLE2	SERPINI1	
KCNB1	MCPH1	NDUFS8	PEX12	PROC	SETBP1	
KCNC1	MDGA2	NDUFV1	PEX13	PRODH	SGCE	


Gene	Gene	Gene	Gene	Gene	Gene	
SGSH	SLC25A22	SOS1	SYN1	TPP1	UNC80	
SHANK3	SLC26A4	SPAST	SYN2	TREM2	VPS13A	
SHH	SLC2A1	SPTAN1	SYNGAP1	TREX1	VPS13B	
SHOC2	SLC35A1	SPTLC2	SYNJ1	TRPM6	VRK1	
SIK1	SLC35A2	SRD5A3	SYP	TSC1	VRK2	
SIX3	SLC35C1	SRPX2	SZT2	TSC2	VWF	
SLC12A5	SLC46A1	ST3GAL2	TACO1	TSEN2	WDR45	
SLC13A5	SLC6A1	ST3GAL3	TBC1D24	TSEN34	WWOX	
SLC16A2	SLC6A8	ST3GAL5	TBP	TSEN54	XK	
SLC17A5	SLC9A6	STRADA	TBX1	TUBA1A	YWHAG	
SLC19A3	SLC9A9	STS	TCF4	TUBG1	ZDHHC15	
SLC1A2	SMC1A	STX1B	TH	TUBGCP6	ZEB2	
SLC1A3	SMPD1	STXBP1	TMEM165	TUSC3	ZFYVE26	
SLC20A2	SMS	SUCLA2	TMEM216	TWNK	ZNF41	
SLC25A12	SNIP1	SUMF1	TMEM67	TYROBP		
SLC25A15	SNRPN	SUOX	TMEM70	UBA5		
SLC25A19	SOBP	SURF1	TNK2	UBE3A		
